# Displaced Myonuclei in Cancer Cachexia Suggest Altered Innervation

**DOI:** 10.3390/ijms21031092

**Published:** 2020-02-06

**Authors:** Nissrine Daou, Medhi Hassani, Emidio Matos, Gabriela Salim De Castro, Raquel Galvao Figueredo Costa, Marilia Seelaender, Viviana Moresi, Marco Rocchi, Sergio Adamo, Zhenlin Li, Onnik Agbulut, Dario Coletti

**Affiliations:** 1 Sorbonne Université, Institut de Biologie Paris-Seine (IBPS), CNRS UMR 8256, Inserm ERL U1164, Biological Adaptation and Ageing, F-75005 Paris, France; daou.nissrine@gmail.com (N.D.); medhi.hassani@uniroma1.it (M.H.); zhenlin.li@sorbonne-universite.fr (Z.L.);; 2 DAHFMO Unit of Histology and Medical Embryology, Sapienza University of Rome, 00161 Rome, Italy; viviana.moresi@uniroma1.it (V.M.); sergio.adamo@uniroma1.it (S.A.); 3 Interuniversity institute of Myology, 00161 Rome, Italy; 4 Department of Physical Education, Federal University of Piauí, Teresina 64000-805 PI, Brazil; 5 Institute of Biomedical Sciences, University of Sao Paulo, Sao Paulo 05508-000 SP, Brazil; 6 Department of Biomolecular Sciences, University of Urbino Carlo Bo, 61029 Urbino, Italy; marco.rocchi@uniurb.it

**Keywords:** Central nuclei, muscle regeneration, striated muscles, c26-colon carcinoma, cancer cachexia, altered innervation

## Abstract

An idiopathic myopathy characterized by central nuclei in muscle fibers, a hallmark of muscle regeneration, has been observed in cancer patients. In cancer cachexia skeletal muscle is incapable of regeneration, consequently, this observation remains unaccounted for. In C26-tumor bearing, cachectic mice, we observed muscle fibers with central nuclei in the absence of molecular markers of bona fide regeneration. These clustered, non-peripheral nuclei were present in NCAM-expressing muscle fibers. Since NCAM expression is upregulated in denervated myofibers, we searched for additional makers of denervation, including AchRs, MUSK, and HDAC. This last one being also consistently upregulated in cachectic muscles, correlated with an increase of central myonuclei. This held true in the musculature of patients suffering from gastrointestinal cancer, where a progressive increase in the number of central myonuclei was observed in weight stable and in cachectic patients, compared to healthy subjects. Based on all of the above, the presence of central myonuclei in cancer patients and animal models of cachexia is consistent with motor neuron loss or NMJ perturbation and could underlie a previously neglected phenomenon of denervation, rather than representing myofiber damage and regeneration in cachexia. Similarly to aging, denervation-dependent myofiber atrophy could contribute to muscle wasting in cancer cachexia.

## 1. Introduction

Skeletal muscle fibers are syncytial in nature, containing hundreds of myonuclei positioned at the periphery of each myofiber in a non-random position, which minimizes transport distances between the nuclei themselves and the other regions of the myofiber [1,2]. Muscle fiber hypertrophy is accompanied by the addition of nuclei from stem cells, while the possible loss of nuclei following atrophy is still controversial [3]. In his seminal work, Spiro noted mispositioned myonuclei in a patient with myotubular myopathy, one of the central nuclear myopathies (CNM) [4]. These mispositioned myonuclei are referred to as centrally located/positioned nuclei or as central nuclei [5]. Central nuclei are a prominent feature of many muscle disorders and they are considered a consequence of myofiber regeneration, as in muscular dystrophy [6].

In muscle fibers the peripheral, subsarcolemmal position of myonuclei, typical of physiological conditions in adults, is not achieved through a straightforward process. During muscle development, mono-nucleated myoblasts fuse to form multi-nucleated myotubes, i.e. nascent muscle fibers [7]. The latter mature into myofibers by fusing with each other and with additional myoblasts. At this stage, myonuclei are in a central position. The maturation of myofibers is characterized by the development of a dense myofibril network filling the cell and by a process of nuclear movement to the periphery of the myofiber [7]. In adult life, similar nuclear movements are seen during myofiber repair, following the incorporation of new myonuclei deriving from satellite cells into the damaged myofibers [8,9]. The newly incorporated nuclei are moved to the center of the myofiber before being moved back to the cell periphery, rather than assuming this position immediately [10], suggesting that there is a biological need for these long-range nuclear movements in muscle development and repair. It is important to understand the mechanisms that drive these nuclear movements and their biological significance to treat muscle diseases involving myonuclear alterations. So far, it has been established that microtubules and other cytoskeletal components play a major role in nuclear positioning [11], and that their perturbation leads to impaired muscle function [12].

Cancer cachexia (CC) is a syndrome characterized by the loss of skeletal muscle mass leading to progressive muscle functional impairment [13,14]. Muscle wasting is the most important phenotypic and clinical feature of CC and, at least in part, is accounted for by impaired skeletal muscle regeneration [15,16]. Worth noting, chemotherapy, used against cancer, induces cachexia and muscle wasting per se [17]. We and others have shown that cachexia is characterized by increased inflammatory markers in the skeletal muscle and by satellite cell activation [18,19]. However, in cachexia satellite cells fail to fuse with muscle fibers and their impairment contributes to muscle wasting [20,21,22]. Recently, Cui and al. described abnormalities in nuclear positioning within myofibers in a non-human primate (NHP) radiation-induced cachexia model, suggesting that the muscle was undergoing degeneration–regeneration processes [23]. Another study in cancer patients reported a myopathy characterized by central nuclei [24]. However, since cachexia and pro-inflammatory cytokines hamper muscle regeneration [20,25,26], finding central nuclei in muscle fibers of cachectic patients and animals is unaccounted for, given that central nuclei do not belong to bone fide regenerating fibers in this condition.

Sarcopenia is another form of muscle atrophy, which shares some features with muscle wasting observed in cachexia, whilst being more progressive and associated to aging rather than to chronic diseases [27]. One of the proposed mechanisms for the atrophy of individual fibers in aging is the underlying loss of motor-neurons [28]. Age-related neuromuscular junction degeneration has been associated to muscle stem cell and myonuclear loss [29]. Indeed, denervation following spinal cord or nerve injury is characterized by displaced myonuclei and by a profound muscle fiber atrophy [30].

To the best of our knowledge, denervation or altered neuro-muscular junctions have never been described in cachexia. Some evidence indirectly suggests that this phenomenon occurs in cachexia, but it has been neglected so far: a) a functional deficit, suggestive of altered neuro-muscular junctions, is observed in both aging and cachexia; b) in aging denervation affects fast fibers, and muscle fiber atrophy preferentially occurs in fast fibers in both aging and cachexia; c) similarly to what happens upon nerve injury, displaced myonuclei have been observed in the musculature of cancer patients. Consequently, if the central nuclear position were solely due to muscle fiber regeneration, this abnormal position in cachectic muscle in the absence of muscle regeneration is a contradiction to the current knowledge of muscle physiopathology.

To address these issues, we hypothesized that denervation phenomena occur in cachexia and contribute to fiber atrophy, accounting for the rare but consistent altered position of myonuclei in a subset of the muscle fibers in both cachectic humans and mice. To show that the central nuclei in cachectic muscle may not be a consequence of regeneration but a sign of denervation, we analyzed the skeletal muscle of both cancer patients and tumor-bearing mice in search of morphological and molecular markers of muscle denervation.

## 2. Results

### 2.1. Muscle Fibers with Central Nuclei in Adults do not Necessarily Show Markers of Regeneration

**Figure 1:**
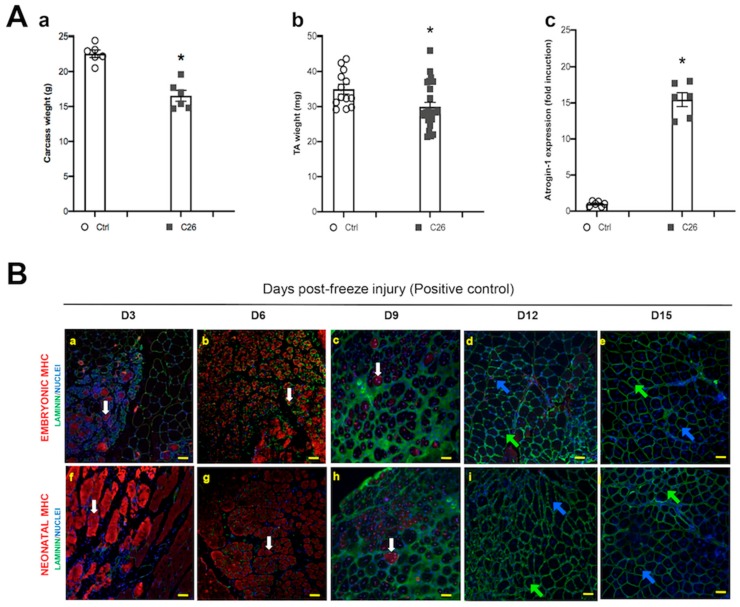

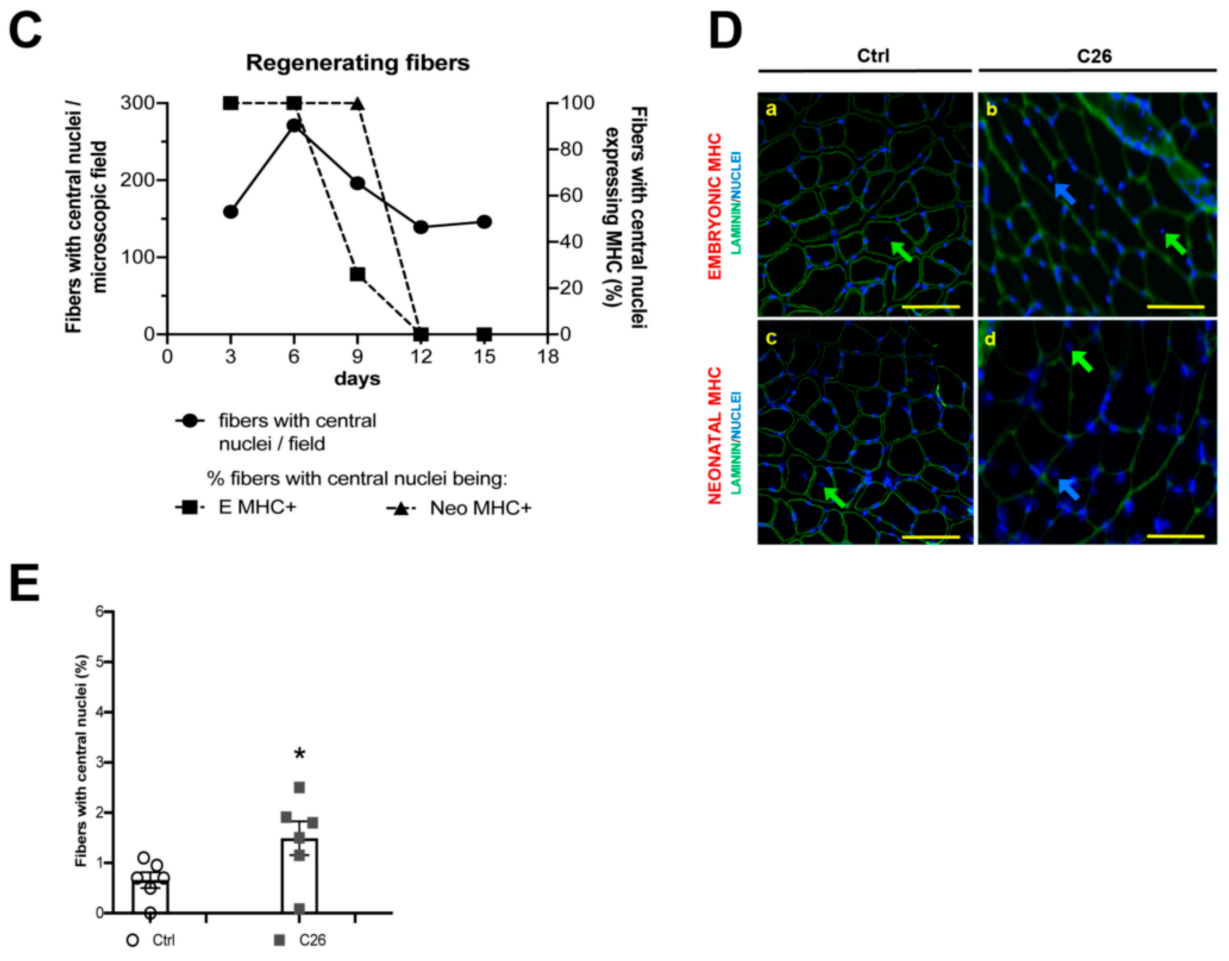
Myofibers with central myonuclei do not express bona fide early regeneration markers in the tibialis of cachectic mice. (**A**) Carcass (i.e. net body weight, minus tutor weight) (**a**) and tibialis anterior (TA) muscle (**b**) weight in control (Ctrl) and in tumor-bearing (C26) mice, showing a significant decrease of body and muscle mass in cachexia. Muscle wasting is due to protein dismantling, as shown by the muscle-specific Ub-ligase Atrogin-1 upregulation in cachexia (**c**). For carcass weight (**a**) and Atrogin-1 (**c**) n = 6 for each group; for TA weight n = 12 and n = 25, for Ctrl and C26, respectively; * *p* < 0.05, by Student’s t test. (**B**) Photomicrographs of TA muscle at 3, 6, 9, 12 and 15 days (D3 to D15) following freeze injury, immunostained for embryonic (**a**–**e**) or neonatal (**f**–**j**) MHC (red); nuclei are counterstained by Hoechst (blue), while laminin immunostaining (green) highlights muscle fibers. Embryonic MHC+ and MHC- fibers with central myonuclei are indicated by white arrows (**a**,**b**,**c**,**f**,**g**,**h**) or blue and green arrows (**d**,**e**,**i**,**j**), respectively. (**C**) Quantification of muscle fibers showing central myonuclei and expressing embryonic MHC. These fibers stop expressing the regeneration markers at D9. (**D**) Photomicrographs of TA muscle immunostained for embryonic (**a** and **b**) or neonatal (**c** and **d**) MHC (red) and laminin (green), in control (**a**, **c**) and C26-tumor bearing (**b**, **d**) mice; nuclei are counterstained by Hoechst (blue). Fibers with central myonuclei, negative for either type of MHC, are indicated by blue or green arrows (in the first case the nucleus is just displaced from its subsarcolemmal position, while in the second case it is closer to the center of the fiber). Scale bar is 50 μm. (**E**) Quantification of the fibers with central myonuclei observed in D.

In order to demonstrate that overt cachexia was occurring in our experimental settings, a well-established model of cancer cachexia was used, i.e. the C26 colon carcinoma-bearing mice [31]. Indeed, tumor-bearing mice showed a marked decrease in body weight as compared to healthy controls (Figure 1Aa); consistent with the expected decrease in muscle mass, the weight of the tibialis anterior (TA) muscle diminished by about 15% as compared to controls (Figure 1Ab). These observations were also confirmed by the upregulation of the muscle-specific, Ub-ligase Atrogin-1 in the cachectic animals (Figure 1Ac).

Muscle damage typically occurs in cachexia [20]. Since central nuclei are expected following muscle damage, we investigated the correlation between altered nuclear position and the expression of molecular markers of muscle regeneration. As a positive control for muscle regeneration we used regenerating TA at different time points following freeze injury and found fibers with central nuclei, as well as the expression of the embryonic and the neonatal isoforms of MHC as expected [32] (Figure 1B). In particular, the number of muscle fibers with central nuclei peaked at day 6 following injury and remained stable in the time-frame analyzed (3–15 days following injury, Figure 1C). On the same sections, we also quantified the percentage of muscle fibers with central nuclei, which were also expressing regeneration markers, such as the embryonic or the neonatal MHC, and we noticed that while the totality of nascent regenerating fibers expressed these markers, the number of fibers with central nuclei (i.e. still regenerating) expressing perinatal MHC dropped to zero by day 12 following injury (Figure 1C). This finding indicates that the expression of pre- or peri-natal MHC isoforms can be detected in all regenerating fibers at early stages of regeneration (3–6 days) and lasts at least 9 days after damage; incidentally, we noted that central nuclei are a more persistent feature of regenerating fibers as compared to MHC.

The same analysis to the TA from control and cachectic mice was applied in the absence of an experimentally induced freeze injury. In both groups we observed rare muscle fibers with central nuclei and a modest but significant increase of central myonuclei in the TA of C26-bearing mice (Figure 1D,E). However, none of these fibers showing altered nuclear positioning was expressing embryonic or neonatal MHC (Figure 1D), suggesting that if they were regenerating fibers they would be “older” than couple of weeks at the time of the analysis (d19 of tumor burden), based on our positive controls (Figure 1B,C). Since this possibility looked unlikely (increased muscle damage had to be occurring during the first week of tumor burden, when the negative effects of the tumor are not occurring, yet), we decided to test the hypothesis that central nuclei could arise in the absence of regeneration phenomena.

### 2.2. Central Nuclei are Clustered in Association with Neurological Perturbations in Cachexia

**Figure 2:**
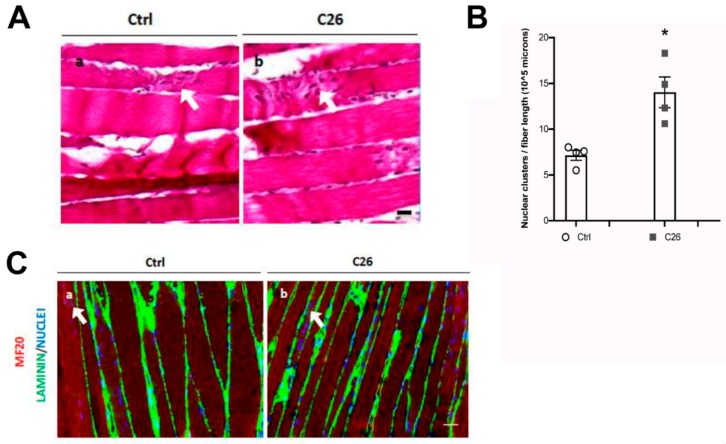

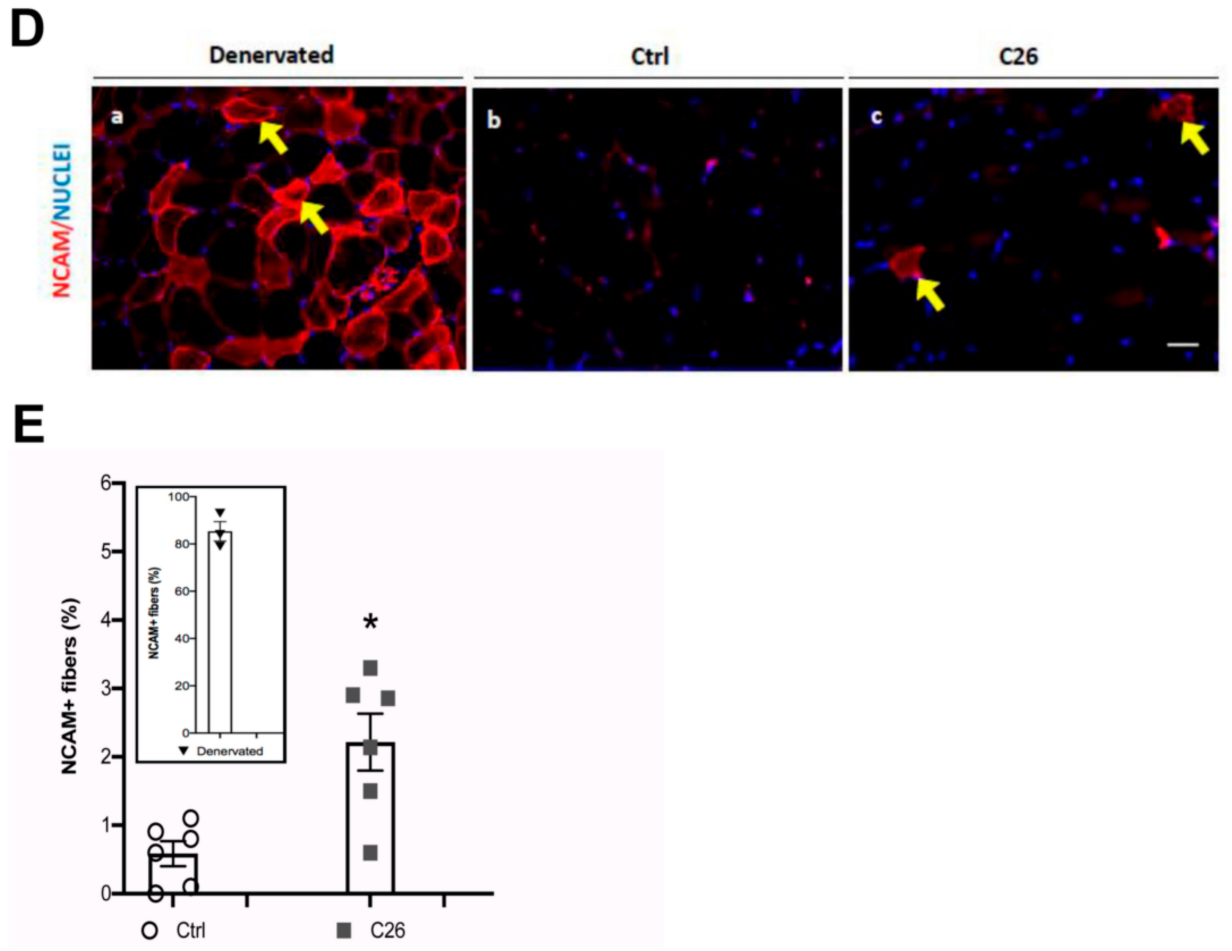
Central myonuclei are clumped in atrophic myofibers and their presence is associated to N-CAM upregulation in the tibialis of cachectic mice. (**A**) Photomicrographs of H&E-stained longitudinal sections of TA muscle, showing clumps of nuclei (arrows) in both healthy (a, Ctrl) and in cachectic (b, C26) muscles; however, in the latter, the frequency of clumps is significantly increased, as shown in the corresponding quantification (**B**); n = 6 for each group, * *p* < 0.05 by Student test. (**C**) Immunofluorescence analysis for MHC (red), laminin (green) and nuclei (blue) showing the occurrence of nuclear clumps (arrow) within the myofibers and not in the interstitial space, in longitudinal sections of the TA muscle. (**D**) Immunofluorescence analysis for N-CAM (red), nuclei are counterstained by Hoechst (blue) on transversal section of TA muscle. Fibers expressing high levels of N-CAM (arrows) are numerous in denervated muscles (**a**, positive control), rare in controls (**b**) and significantly higher in cachectic muscles (**c**). NCAM is also detectable in satellite cells, which are distinguishable as dots. Quantification of the percentage of N-CAM+ fibers is shown in (**E**), for control (Ctrl), cachectic (C26) and denervated (inset) muscles. Data are presented as mean +/- SEM, *n* = 6 for each group. Scale bar is 10 μm in panel A, 25 μm in panel C and D.

With the aim to better characterize the nature of central myonuclei in cachexia, we further analyzed murine TA in longitudinal sections, which specifically allow the observation of nuclear clusters or clumps (Figure 2A). Preliminarily, we noticed a statistically significant difference in the average fiber length that we were able to measure on a single random longitudinal section of muscle in healthy and cachectic mice (274 ± 26 and 193 ± 16 micron for healthy and cachectic muscle, respectively). This observation that cachectic fibers are less straight and parallel than healthy fibers, suggests that cachectic muscle could be more disorganized than healthy muscle. In addition, we observed central nuclei forming clusters, in 7% and 14% of the myofibers from control and C26-bearing mice, respectively, and that their number was significantly increased in cachectic muscles (Figure 2B). The fact that clustered nuclei were not simply belonging to inflammatory infiltrate but were actually myonuclei was demonstrated by a co-staining for MHC showing that central nuclei were located within the muscle fibers and not in laminin tubes in regions between clamps of broken myofibers (Figure 2C). Since nuclear clumps are a hallmark of denervated myofibers, these abnormal morphological aspects suggested that the nuclear redistribution may have been the result of denervation phenomena.

To confirm the occurrence of neurological perturbations in cachexia, we also immunostained the murine TA for NCAM, a marker of denervated myofibers, also expressed in satellite cells [30]. As a positive control, NCAM expression was confirmed in 86 ± 4% of muscle fibers upon sciatic nerve resection (Figure 2Da). When we extended the analysis to control and cachectic muscles, we noticed a significant increase in the number of NCAM-expressing muscle fibers, but not of NCAM-expressing satellite cells, in cachexia (0.6 ± 0.2% and 2.2 ± 0.4% in control muscle and cachectic muscle, respectively; Figure 2Db, 2Dc and 2E). Taken together, these observations suggested that in cachexia nuclear displacement is associated with altered innervation or with a perturbation of the crosstalk between muscle fibers and their innervating motor neurons.

### 2.3. Tumor-Bearing Mice Show Signs of a Myopathy Associated with the Upregulation of Denervation Markers

**Figure 3:**
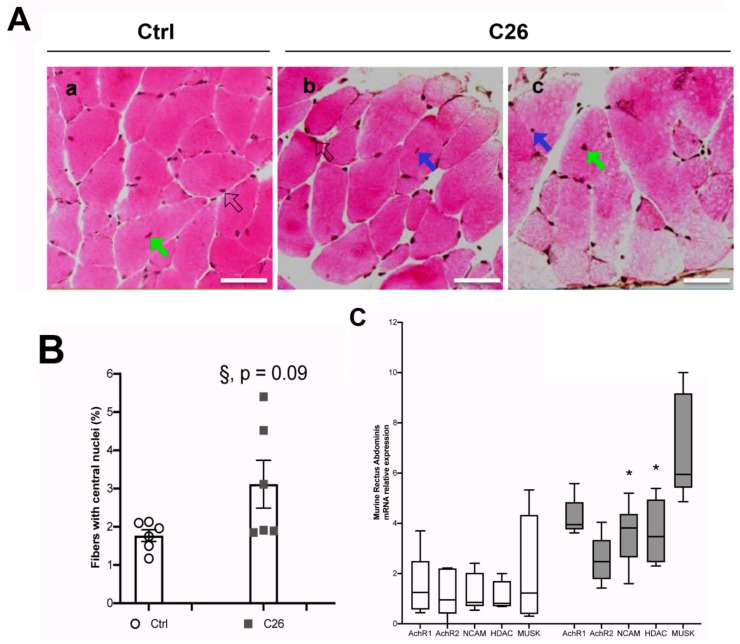
An increased number of central nuclei in the murine Rectus Abdominis (RA) fibers is associated with cachexia and denervation markers. (**A**) Representative images showing H&E staining of cross sections from the RA of control (**a**, Ctrl) and C26-tumor bearing (C26) mice; for the latter, two panels from different regions of the muscle are shown (**b** and **c**). Central nuclei are indicated by blue or green arrows (in the first case the nucleus is just displaced from its subsarcolemmal position, while in the second case it is closer to the center of the fiber); peripheral (i.e. normal) myonuclei are indicated by open arrows. Scale bar is 50 μm. (**B**) Percentage of fibers with central nuclei in control (Ctrl) and C26 mice. The Student’s t test showed a quasi-significant difference (§, p = 0.09) between Ctrl and C26. (**C**) Q-PCR analysis on muscle from the RA of Ctrl and C26 mice for denervation markers as indicated. Data are presented as box-and-whisker plot, showing the median +/- 10–90 percentile range, and analyzed by using Wilcoxon-Mann-Whitney test; * *p* < 0.05; *n* = 6 for each group.

With the aim of confirming the occurrence of morphological alterations related to nuclear mispositioning in a different muscle type, we extended our analysis to the rectus abdominis (RA) muscle of control and cachectic mice (Figure 3A). We quantified the number of muscle fibers with central nuclei in cross sections of the RA, and found a quasi-significant difference by comparing controls and C26 cachectic mice (*p* = 0.09; Figure 3B). However, we noticed that muscle fibers with central nuclei were rare (1–3% of the muscle fibers in cross-sections) and could not exclude a false negative result. Indeed, by using a more global approach (i.e. muscle lysates) we were able to demonstrate a significant upregulation of denervation markers in the muscle in C26 mice as compared to controls (Figure 3C). The expression of several genes—Acetylcholine receptors (AchR), N-Cell Adhesion Molecule (N-CAM), Histone Deacetylase 4 (HDAC4), and MUScle associated receptor tyrosine Kinase (MUSK)—is known to increase in denervated fibers. We used the Wilcoxon-Mann-Whitney test to individually assess each gene and we found a statistically significant increase of NCAM and HDAC expression in the cachectic muscle with respect to the control (Figure 3C). Taken together these findings show that the increase in central myonuclei in cachectic muscle is associated with an increase in several genes known to be upregulated in denervation, which is consistent with a small, but significant amount of muscle fiber denervation contributing to muscle wasting.

### 2.4. Cancer Patients Show Signs of a Myopathy Associated with the Upregulation of Denervation Markers in Overt Cachexia

**Figure 4:**
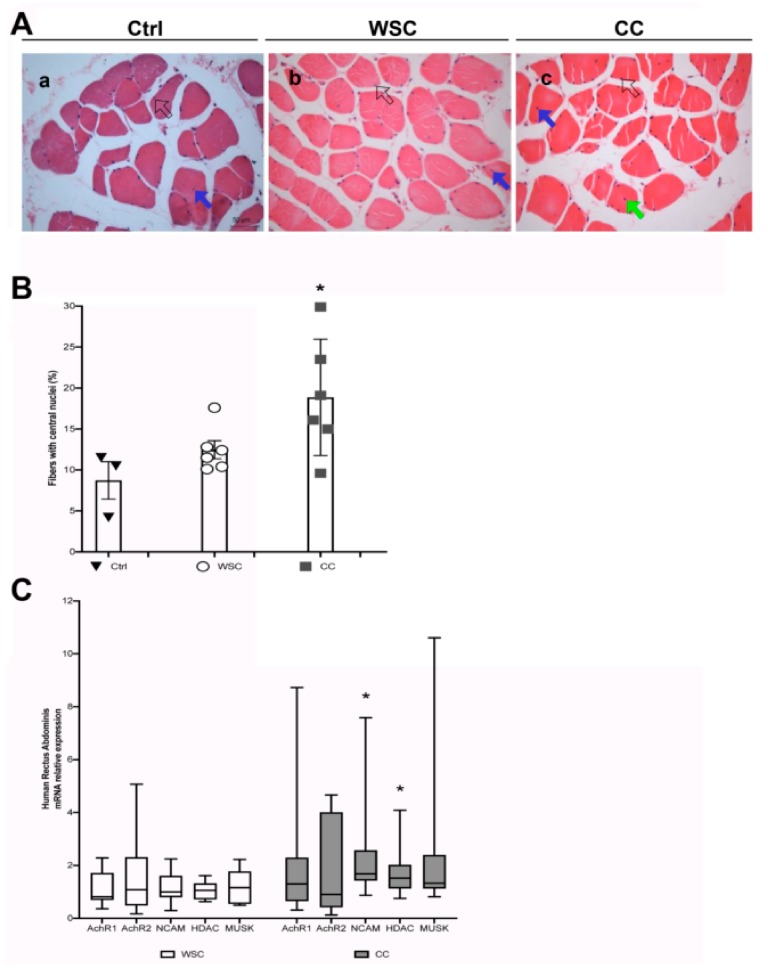
An increased number of central nuclei in the human Rectus Abdominis fibers is associated with cachexia and denervation markers. (**A**) Representative images showing H&E staining of cross sections from the RA muscle of patients without cancer (**a**, Ctrl), Weight-Stable Cancer patients (**b**, WSC) and Cancer Cachexia patients (**c**, CC). Central nuclei are indicated by blue or green arrows (in the first case the nucleus is just displaced from the subsarcolemmal position, while in the second case it is closer to the center of the fiber); peripheral (i.e. normal) myonuclei are indicated by open arrows. Scale bar is 50 μm. (**B**) Percentage of fibers with central nuclei in muscle cross-sections of Ctrl, WSC and CC patients (a randomly chosen subset of the patients in Table 1). The three groups showed significant differences in the number of fibers with central myonuclei (F = 4.93; df = 2; p = 0.025 by ANOVA; * *p* < 0.05 by Tukey’s HSD test, used as a post-hoc test for CC vs Ctrl). Data are presented as mean +/- SEM, n = 3–6 for each group. (**C**) Q-PCR analysis on muscle from the RA of WSC (n = 10) and CC (n = 13) patients for denervation markers as indicated. Data are presented as box-and-whisker plot, showing the median +/- 10–90 percentile range, and analyzed by using Wilcoxon-Mann-Whitney test; * *p* < 0.05.

To validate the previous findings in the animal model of cancer cachexia, we analyzed muscle biopsies from the RA of cancer patients (whose clinical features are shown in Table 1). These were taken during abdominal surgery of patients suffering from gastrointestinal cancers. We quantified the percentage of fibers showing central nuclei in cross-sections, and compared control patients (healthy subjects) with cancer patients displaying weight loss (CC, cachectic cancer) or failing to display it (WSC, weight stable cancer patients; Figure 4A). We found a progressive, statistically significant increase in the number of fibers showing central nuclei starting from healthy subjects, to WSCs and CCs: the actual increase in WSC and CC patients was about 34% and 50%, respectively, as compared to patients without cancer (Figure 4B). The ANOVA showed that the amount of central myonuclei was affected by the patients’ status; in addition, Tukey’s post-hoc test showed a statistically significant difference between CC and control patients, but not between WSC and control patients, indicating that the doubling in the number of fibers displaying central nuclei was closely associated with the onset of overt cachexia. We hypothesized that in these muscles the same changes and neurological perturbations observed in mice can occur. To test the expression of the same set of denervation markers as above, we used the Wilcoxon-Mann-Whitney test to individually assess each gene in the CC as compared to WSC patients and we found a statistically significant increase of NCAM and HDAC expression in the cachectic muscle with respect to the muscle of weight stable patients (Figure 4C).

## 3. Discussion

We observed an increase in denervation markers in cachectic muscle, occurring in association with clustered, central myonuclei. Our data in both cancer patients and tumor-bearing mice indicate the existence of previously unobserved neurological perturbations which are specifically associated with the onset of cachexia.

Since the seminal studies by Veratti and others, the peculiar, syncytial nature of muscle fibers has driven research interest toward their nuclei [33]. These occupy a typical, subsarcolemmal position in the myofiber. The evolutionary reason for this position is still unknown, even though it can be speculated that this is the only site which does not interfere with the contractile activity of the myofibrils packed in the myofiber center. In rodents, myonuclei are regularly spaced longitudinally, at a distance of 100–150 microns form each other [34], an observation that led to the general idea of myonuclear domain [35]. This may be related to the lack of a long-distance transport system for proteins within the myofiber, which is among the largest cell types. Each nucleus maintains the control of a region of the muscle fiber—a concept referred to as nuclear domain [36]. Damaged myofibers are repaired by the activation of satellite cells, which ultimately fuse with each other and with muscle fibers, adding their nuclei to the syncytium at the end of the process [8,9]. The newly incorporated nuclei are moved to the center of the myofiber before being moved back to the cell periphery, rather than assuming this position immediately [10]. This seems to clash with the best usage of energy made by organisms and cells, suggesting that there is a biological need for these long-range nuclear movements in muscle development and repair.

The mechanisms controlling the position of nuclei in skeletal muscle fibers are only partially known. Nuclear movement and positioning in skeletal muscle fibers are controlled by a complex system involving all three cytoskeletal components (microfilament, intermediate filaments and microtubules) and responsible for nuclear centration, spreading, dispersion, and clustering [10]. Tubulin and dynein allow nuclear movements, while actin and desmin collaborate in anchoring myonuclei [10,37,38]. In addition, the desmin-based muscle intermediate filaments are responsible for both myonuclear orientation in parallel to the longitudinal axis and for their regular distribution along the myofiber [34]. Since actin-binding protein Syne-1, which spans the nuclear membrane, is a nuclear anchor, its absence causes the dispersion of the nuclei clustered at the level of the NMJ [39]. In addition, the link between the nucleus and the cytoskeleton affects nuclear rotation, myogenesis and mechanotransduction [40], which, in its turn, activates transcription factors that are essential for muscle survival and healthy functioning (such as the Serum Response Factor) [41]. Consequently, nuclear mispositioning can cause muscle development defects and diseases.

In pathological conditions myonuclear positioning can be deeply altered, thus taking on a significant value as a morphological marker, testified by the inclusion of nuclear position in clinical guidelines for diagnosis [42]. An alteration in myonuclear positioning is a hallmark of some muscle diseases [43]. Moreover, central nuclei are present in the acute condition represented by muscle regeneration following injury. Whilst minimal basal muscle damage and regeneration appear to occur continuously in mammals (1% of the myofibers in the Soleus) [44], massive muscle regeneration follows the inflammatory phase subsequent to chronic or acute muscle damage [8,22]. This is the only case (sarcoplasm fragmentation and inflammatory cell infiltration) in which regeneration occurs *strictu sensu*, as pointed out by Grounds [45]. Additionally, the association of central nuclei with embryonic myosin expression are not unique to muscle regeneration [46]. In this regard we found that in cachexia central nuclei are not limited to regenerating myofibers.

In addition to muscle-specific damage, another pathological condition leading to myonuclear misplacement and severe myofiber atrophy is denervation due to spinal cord injury or motor neuron toxicity. Myofibers survive denervation, but they show a typical nuclear misplacement consisting in nuclear clumps followed by stretches of anucleated (and amyofibrillar) sarcoplasm [30,47,48]. Denervation is also characterized by the specific upregulation of several molecular markers, including AchRs and Musk (which are involved in the NMJ) and NCAM and HDAC [46,49].

We are reporting here a small, but significant increase in muscle fibers containing central nuclei in the muscle of both cachectic human patients and mice. We also observe a significant upregulation of several molecular markers of denervation, in the absence of any molecular evidence of ongoing muscle regeneration, as in muscle injury. Our observations strongly suggest that altered innervation or motor neuron death occur in cachexia. This would be consistent with the indirect evidence of altered motor nerve conduction velocity reported for patients with anorexia nervosa, a condition leading to cachexia [50]. In line with a previous study on humans [24], we also observe N-CAM and HDAC upregulation in atrophying fibers in both cachectic patients and tumor-bearing mice. This strong indication of denervation is associated with altered nuclear positioning, namely the occurrence of nuclear clumps in tumor-bearing mice and the significant increase of central nuclei in both cachectic patients and mice. To the best of our knowledge, this is the only direct evidence for any nerve alteration associated with cachexia. The number of muscle fibers affected by this phenomenon is relatively small in cachectic mice (up to about 3% of fibers with central nuclei, and to about 2% of NCAM-expressing fibers as compared to 86% of NCAM-expressing fibers in a fully denervated muscle). This suggests the occurrence of focal denervation phenomena or local alterations of the NMJ in a limited number of fibers. Worth noting, the experiments in mice last a few weeks only, while humans are analyzed several weeks following the tumor onset, and indeed the phenomenon in humans is one order of magnitude bigger (about 20% of fibers with central nuclei in cachectic patients) and, therefore, definitely significant from a pathophysiological point of view.

The low number and the scattered nature of the myofibers showing displaced nuclei—sometimes grouped in clusters of two to five fibers—mirrors a similar phenomenon in sarcopenia which is due to single motor neuron loss [51]. Even though aging-associated sarcopenia and cachexia display different histological features [52], motor unit alterations could be a novel shared feature of these two forms of muscle atrophy. An alteration of muscle fiber innervation contributes to explaining the functional deficit observed in cachectic muscles. It is well known that cachectic mice and men have lower muscle performance and a high prevalence of fatigue [53,54]. Similar functional deficits are reported in sarcopenia: focal denervation accounting for local switches to a slow fiber phenotype, severe atrophy of certain muscle fibers, and diminished muscle force [55]. Our findings could be useful to explain why physical activity spontaneously declines in cancer patients [56] and why making cancer patients exercise is not a straightforward endeavor. It would be of the greatest interest to investigate whether exercise prevents the observed NMJ dysfunction and related muscle fiber alterations. In addition, our observations on muscle denervation in cachexia could explain a previously reported subclinical myopathy in cancer patients, characterized by central nuclei in myofibers [24]. Since muscle regeneration is strongly inhibited in cachexia [20,57,58,59], the central nuclei in cachectic muscles were so far unaccounted for, while our proposed model based on denervation could provide and explanation for this phenomenon (graphical abstract).

Based on all of the above, we propose a model whereby the increased frequency of displaced myonuclei observed in cachexia is explained by denervation or an alteration in the NMJ, rather than to myofiber regeneration. This choice is supported by the following considerations: 1) even though we and others reported muscle damage in cancer cachexia, muscle regeneration is severely hampered in this condition [20,54,57]; therefore the occurrence of central nuclei due to myofiber neoformation or repair following damage is unlikely; 2) the kinetics of myofiber regeneration is not compatible with our observations of displaced nuclei existing in the absence of perinatal myosins. Since we observed displaced nuclei on day 19 following tumor transplantation and in the absence of regeneration markers, we believe two scenarios are possible: either central nuclei persisted in the myofibers following damage which occurred exclusively between day 1 and day 10 following tumor transplantation, or central nuclei appeared independently of fiber damage.

## 4. Methods

### 4.1. Mice and Tumor Transplant

Cachexia was induced by subcutaneous grafting of a 0.5 mm^3^ fragment of colon carcinoma (C26, originally obtained from the ATCC) in the dorsal region of female BALB/c AnNJ mice, aged 10 weeks (Janvier Labs, Le Genest-Saint-Isle, France). All experiments were performed in accordance with the guidelines of the Institutional Animal Care and Use Committee (authorization by the Ethical Committee C. Darwin, Paris: project #1944, 1 Feb 2017) and with national and European legislation (Directive 2010/63/EU). Three weeks after tumor grafting, the tibialis anterior (TA) muscle and the rectus abdominis (RA) muscle were dissected and analyzed. Muscles of sham transplanted mice were used as controls. A regenerating TA, following focal injury, or 1-week sciatic nerve resection, was used as positive control for some experiments.

### 4.2. Histology and Immunofluorescence Analyses

Muscles were frozen in liquid nitrogen-cooled isopentane. Muscle crysections (either transversal or longitudinal sections of 8 μm thickness) were fixed in 4% formaldehyde in PBS for 5 min and either stained with hematoxylin and eosin (H&E) for morphometric analysis or processed for immunofluorescence analysis. Primary antibodies: rabbit anti-laminin Ab diluted 1:100 (Sigma – Aldrich, St Louis, MO, USA) in 2% BSA/PBS overnight at 4 °C; anti-embryonic Myosin Heavy Chain (MHC) Ab (clone F1.652) diluted to 1:20; anti-neonatal MHC Ab diluted 1:20 (clone N1.551), or with MF20 Ab diluted 1:20 (clone MF20). Monoclonal antibodies were obtained from the Developmental Studies Hybridoma Bank, created by the NICHD of the NIH and maintained at the University of Iowa. Secondary antibodies: Fluor 488-conjugated goat anti-rabbit antibody; Fluor 596-conjugated goat anti-mouse antibody (Invitrogen Molecular Probes, Carlsbad, CA, USA), both diluted 1:400 in 2% BSA/PBS. The sections were mounted with fluoromount-G (Biotech, Paris, France). Nuclei were visualized by Hoechst staining. Photomicrographs were obtained by means of a Leica DMi8 microscope fitted with a Leica DiM8 camera.

### 4.3. Patients’ Characteristics

Patients with gastrointestinal cancer (n = 23, further subdivided into weight stable and cachectic patients, see below) and in non-neoplastic conditions (*n* = 3) were recruited at the University Hospital of the University of São Paulo (study approved by University Hospital and Biomedical Sciences Institute São Paulo University Ethics Committees; numbers CEP-HU/USP-752, CAAE:0031.0.198.019.07, ICB 788/07). At the time of assessment, no patient was taking anti-inflammatory drugs or received chemo or radiotherapy previous to tumor resection surgery. Cancer patients (*n* = 23) were divided into 2 groups: Weight Stable Cancer (WSC, n = 10) and Cachectic Cancer (CC, *n* = 13) patients, based on the classification in the paper entitled “Cachexia a new definition” [13]. The clinical data of all these patients and the healthy controls are reported in Table 1. For colorectal and gastric cancer patients muscle samples from Rectus Abdominis were obtained during elective surgery, i.e. colectomies or gastrectomies; for control patients samples came from herniorraphies or exploratory laparotomies. Of the three groups, all samples were analyzed by Q-PCR, while a subset of 3 (for the Control) to 6 samples (for the WSC and CC groups) was randomly chosen for histological analysis.

### 4.4. Histological Analysis of Human Muscle

Human muscle samples were fixed in 4% paraformaldehyde, embedded in paraffin and cut into 5 µm sections for histological evaluation. The slides were stained with hematoxylin and eosin and examined by light microscopy (at least five fields for slide). The number of muscle fibers with central nuclei was expressed as the percentage of the total number of myofibers per sample.

### 4.5. Quantitative PCR Analysis

Total RNAs were extracted from muscle tissue by using TRI Reagent (Sigma, St Louis, MO, USA), or TRIzol^®^ (Trizol reagent—Invitrogen, Life Technologies, Carlsbad, CA, USA) for murine or human muscle, respectively, and reverse-transcribed with a high capacity cDNA reverse transcription kit (Applied Biosystems, Foster City, CA, USA) including random hexamers. Gene expression was performed with Fast SYBR green master mix (Fast SYBR® Green Master Mix, Applied Biosystems, Foster City, CA, USA) in a QuantStudio 12K Flex Real-Time PCR System instrument (Applied Biosystems, Foster City, CA, USA). The Primer3 program (frodo.wi.mit.edu/primer3/) was used to select specific primers and the housekeeping gene, GAPDH, was used to normalize expression levels. Gene expression was calculated using the ΔΔCT method. The following Mus musculus primers (Invitrogen, Life Technologies, Carlsbad, CA, USA) were used:

AChR1 for: 5’-CTCTCGACTGTTCTCCTGCTG-3’

AChR1 rev: 5’-GTAGACCCACGGTGACTTGTA-3’

AChR2 for: 5’-GGAGAAGCTAGAGAATGGTCC-3’

AChR2 rev: 5’-CCCACTGACAAAGTGACTCTGC-3’

NCAM for: 5’-AGAGGACGGGAACTCCATCA-3’

NCAM rev: 5’-GAGCGCTCTGTACTTGACCA-3’

Hdac4 for: 5’-GTCTTGGGAATGTACGACGC-3’

Hdac4 rev: 5’-GTTGCCAGAGCTGCTATTTG-3’

Musk for: 5’-GTCCCTCCTCCGTGGTTTTC-3’

Musk rev: 5’-CAGGACTGCATCACACACCT-3’

Primers employed in human rectus abdominis gene expression were:

AChRa1 for: 5’-CAGAGTGCCAGTGAGAAGCA-3’

AChRa1 rev: 5’-AGGCCAGCTGAGCAAAGG-3’

AChRg for: 5’-GTCGATCACAACTGGGGAGG-3’

AChRg rev: 5’-CCGGGCCTTTCTCTAGCTTC-3’ 

HDAC4 for: 5’-CCACCTCACTCCCTACCTGA-3’ 

HDAC4 rev: 5’-CTGTGACGAGGGGTGCTTG-3’

MUSK for: 5’-GGAAGTTGAGGTTTTTGCCAGG-3’

MUSK rev: 5’-AGTGCAGGGTCACAAAGGAG-3’

NCAM1 for: 5’-GTGGGCAGACAGAAAGGACA-3’

NCAM1 rev: 5’-CCATGTGCCCATCCAGAGTC-3’

GAPDH for: 5’-CCTCTGACTTCAACAGCGAC-3’

GAPDH rev: 5’-CGTTGTCATACCAGGAAATGAG-3’

### 4.6. Statistical Analysis

Each set of data for the different types of experiments was first analyzed to verify its suitability for parametric analysis by means of Levene’s test. Based on results (independence among groups, homoscedasticity, and normal distribution), data were analyzed by parametric (ANOVA followed by Tukey’s HSD test, used as a post-hoc test, or Student’s t test) or non-parametric tests (Wilcoxon-Mann-Whitney Test), as appropriate; a *p* value < 0.05 was considered as the threshold for significant differences. Data were graphically depicted as bars showing the mean ± SEM (as well as superimposed dot plots to show the dispersion of the data and the n for each group) or as box-and-whisker plots showing the median +/- 10–90 percentile range, for parametric and non-parametric analyses, respectively. Statistical analysis was performed with the SPSS Statistics software version 25 (IBM, Armonk, NY, USA).

## 5. Conclusions

In conclusion, we are reporting the occurrence of central myonuclei associated with muscle wasting in cancer patients and animal models of cachexia. Since these central myonuclei are not the result of muscle regeneration, our novel observations draw attention to a previously neglected factor contributing to cause muscle wasting in cachexia, that is denervation. As a consequence, we believe that multimodal approaches to combat cachexia should include neurological monitoring and interventions.

## Figures and Tables

**Table 1:** Clinical features of patients included in this study.

	Control	WSC	CC
Number of patients	3	10	13
Gender (M / F)	2 / 1	5 / 5	11 / 2
Age (years)	47.3 ± 8.2	62.3 ± 3.7	63.7 ± 3.7
Height (m)	1.56 ± 0.03	1.64 ± 0.03	1.67 ± 0.02
Weight at diagnosis (kg)	68.2 ± 9.1	65.1 ± 4.4	62.1 ± 3.5
BMI (kg/m^2^)	27.2 ± 3.9	24.1 ± 1.4	22.1 ± 0.9
Weight loss (%)	0 ± 0.5 **	3.4 ± 2.2 *	16.7 ± 3.3
Tumor location (stomach / intestine)	NA	4 / 6	4 / 9
Tumor staging			
I	NA	4	2
II	NA	3	3
III	NA	2	2
IV	NA	1	6

Non-cancer patients, used as controls, and gastro-intestinal cancer patients were enrolled at the University of Sao Paulo hospital among patients undergoing abdominal surgery. Legend: BMI: Body Mass Index; Control: patient without cancer; CC: Cachectic Cancer patient; WSC: Weight Stable Cancer patient. Data are expressed as mean ± SEM. One-way ANOVA followed by Tukey post-test was used. WSC—weight stable cancer patients; CC— cancer cachexia patients. ** *p* = 0.0079; * *p* = 0.0016 vs CC. NA: Not Applicable (tumor were absent in the control population).

## Abbreviations

AchR	acetylcholine receptors
CC	cachectic cancer
CNM	central nuclear myopathies
HDAC	histone deacetylase
NCAM	N-cell adhesion molecule
RA	rectus Abdominis
TA	tibialis anterior
WSC	weight stable cancer
MUSK	muscle associated receptor tyrosine kinase
